# Prevalence of chondromalacia patella according to patella type and patellofemoral geometry: a retrospective study

**DOI:** 10.1590/1516-3180.2021.0206.R2.10012022

**Published:** 2022-09-12

**Authors:** Muhsin Dursun, Murat Ozsahın, Guray Altun

**Affiliations:** I MD, PhD. Physician, Department of Orthopedics and Traumatology, EPC Special Hospital, Adana, Turkey.; II MD, PhD. Physician, Department of Orthopedics and Traumatology, Middle East Special Hospital, Adana, Turkey.; III MD, PhD. Physician, Department of Orthopedics and Traumatology, University of Health Sciences Umraniye Training and Research Hospital, Istanbul, Turkey.

**Keywords:** Patellofemoral pain syndrome, Chondromalacia patellae, Patellofemoral joint, Anterior knee pain, Cartilages, Chondral degeneration

## Abstract

**BACKGROUND:**

The relationships between the morphometric structure of the patellofemoral joint, patella type and chondromalacia patella are still a matter of debate.

**OBJECTIVE:**

To identify the prevalence of chondromalacia patella by determining the patella type and making patellofemoral morphometric measurements.

**DESIGN AND SETTING:**

Retrospective cohort study in an orthopedics and traumatology clinic in Turkey, conducted between June 2017 and November 2019.

**METHODS:**

This study involved 562 knees of 522 patients with anterior knee pain (246 males and 316 females; mean age 46.59 years). The patients were grouped according to presence of chondromalacia patella (group I) or absence of chondromalacia patella (group II). The patella type, lateral trochlear inclination, medial trochlear inclination, trochlear angle, sulcus angle, patellar tilt and Insall-Salvati index were assessed. Group comparisons were made using chi-square tests or Student t tests. The r value was used to determine the magnitude of relationships between pairs of variables.

**RESULTS:**

Among the 562 knees evaluated, 265 (50.71%) presented type I patella, 195 (36.7%) type II, 100 (12.3%) type III and 2 (0.3%) type IV. Group I consisted of 448 knees and group II consisted of 114 knees. Significant differences were found between the groups in terms of age, gender, patella type and lateral inclination angles (P < 0.05).

**CONCLUSION:**

Detecting the patella type and making lateral inclination measurements in patients with anterior knee pain are of great importance for diagnosing suspected chondromalacia patella, particularly in the early degenerative period.

## INTRODUCTION

Among all the joints in the human body, the knee undergoes the earliest degeneration in all age groups. Pathological conditions of the retropatellar joint cartilage are an important reason for anterior knee pain.^1,2^ Chondromalacia patella (CP), one of the most common reasons for anterior knee pain, is a progressive disorder that includes softening of the articular cartilage, fibrillation, thinning, focal swelling, ulcerous formations, chondral defects and subchondral erosive changes. This condition does not entail any complaint specific to cartilage diseases or physical examination. In a study evaluating clinical diagnoses in knee-joint pathological conditions, it was reported that among inner-knee conditions, cartilage diseases were the most difficult to diagnose.

None of the imaging methods available, except specific magnetic resonance imaging (MRI) techniques, are known to help in making the diagnosis, since basic imaging methods of the skeletal system are insufficient for monitoring joint cartilage degeneration.^3^ The purpose of cartilage imaging is to evaluate the integrity of the cartilage surface and the thickness and volume of the cartilage matrix and its relationship with the subchondral bone. Although arthroscopic evaluation is a standard criterion for diagnosing CP, it is not preferred, given that this is an interventional procedure.^4^ Therefore, because MRI provides superior resolution in multiplanar imaging between tissues, it is currently the primary diagnostic method in evaluating joint diseases.^3^

Cartilage quality and degeneration are paramount indicators for diagnosing CP on MRI scans. Moreover, trochlear morphology and patella type play crucial roles in CP. The sulcus angle is used for primary assessment of the morphological structure of the trochlea. However, although the sulcus angle can act as a guide for evaluating the geometry of the femoral trochlea, it represents the trochlear surface geometry and this is insufficient for assessing the medial and lateral trochlear anatomy.

Recent studies have reported that the lateral trochlear inclination (LTI) and trochlear angle measurements are alternatives for evaluating the trochlear geometry. Nonetheless, the results from these studies have varied and the relationship is not yet well documented.^4,5,6,7,8,9^

## OBJECTIVE

The purpose of the current study was to evaluate the prevalence of CP and the relationship between the patella type and patellofemoral morphometric measurements. To evaluate patellofemoral morphology, the patella type, LTI, medial trochlear inclination, trochlear angle, sulcus angle, patellar tilt and Insall-Salvati index were assessed.

## METHODS

All the procedures performed in the current study involving human participants were undertaken in accordance with the ethical standards of the institutional and national research committee as well as with the 1964 Helsinki Declaration and its later amendments or comparable ethical standards. Informed consent was obtained from all individual participants included in this study. The study was approved by the local ethics committee of the same hospital (decision no. 352b; dated March 13, 2019). No approval from the National Ethics Committee was necessary, as this was a non-interventional observational study.

In the current study, 882 knees of 800 consecutive patients with anterior knee pain who came for consultations at a single institution between June 2017 and November 2019 were evaluated retrospectively. The inclusion criteria for the study were as follows: the patients needed to be older than 18 years of age and appropriate imaging needed to be available via our institution’s picture archiving system (PACS). Patients with histories of inflammatory arthritis or knee surgery, anterior knee pain complaints that began more than six months earlier and body mass index higher than 35 kg/m^2^ were excluded from the study. The final study cohort consisted of 562 knees of 522 patients (246 males and 316 females). The patients were divided into two groups according to whether chondromalacia patella was present: patients with CP (CP group; n = 448 knees) and without CP (nonCP group; n = 114).

**Figure 1.**
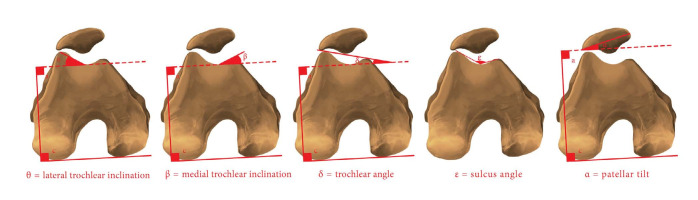
Measurement techniques for patellofemoral joint morphometry values.

The patella type, LTI, medial trochlear inclination, trochlear angle, sulcus angle and patellar tilt were assessed from fat-suppressed proton-density axial and sagittal-fraction MRI scans on all the patients. For the axial fraction measurements, the posteriormost and widest fractions of the medial and lateral posterior condyles were used. Measurements were made as follows: a) with the LTI as the angle between lines passing tangentially to the posterior aspect of the posterior condyles and tangentially to the lateral trochlear facet;^10^ b) with the medial trochlear inclination as the angle between lines passing through the posterior aspect of the posterior condyles and the medial joint surface;^10^ c) with the trochlear angle as the angle between lines passing through the posterior aspect of the posterior condyles and the medial/lateral condyles;^11^ d) with the sulcus angle as the angle between lines passing tangentially to the ventral surfaces of the medial and lateral condyles;^12^ and e) with the patellar tilt angle as the angle between a line passing through the posterior aspect of the posterior condyles and a line joining the lateral and medial edges of the patella^13^ (Figure 1).

**Figure 2.**
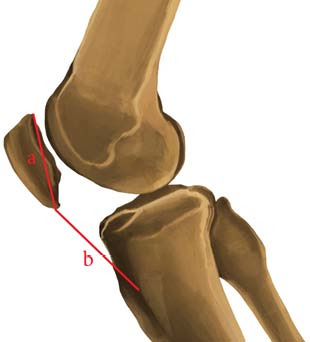
Insall-Salvati index (a/b) measurement method on lateral knee image. a: greatest diagonal length of the patella; b: the length of the patellar tendon (LT) is measured from the patellar apex to the tibial tuberosity.

The Insall-Salvati index was measured using sagittal-fraction MRI to evaluate the location of the patella in the sagittal plane. In areas of the sagittal plane where the Insall-Salvati index was observed to be the highest, the ratio between the longest axis of the patellar tendon and the longest axis of the patella was calculated (Figure 2).^14^ Measurements obtained by two separate specialists were subjected to interobserver testing. The data were distributed and the average of the measurements obtained by these two specialists was used.

The types of patella and CP were evaluated in accordance with Baumgartl’s classification and the Modified International Cartilage Repair Society (ICRS) classification, respectively.^15,16^

**Figure 3.**
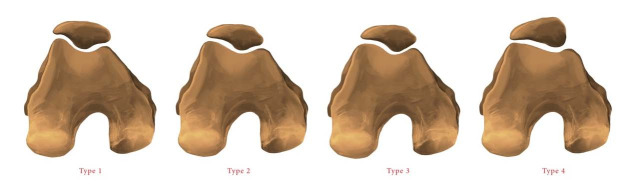
Patella types according to Baumgartl classification: type 1, both facets are equal and concave; type 2, lateral facet is more prominent and medial facet is planar; type 3, medial facet is convex; and type 4. there is no medial facet (“jockey cap”).

Baumgartl’s classification was graded as follows: type I patella has medial and lateral facets that are both concave and of equal length; type II patella has a lateral facet that is more prominent than the medial facet, while the medial facet is plane or concave; type III patella has a smaller and convex medial facet; and type IV patella has no medial facet or central rim and is also referred to as the “jockey cap” (Figure 3).

According to the Modified International Cartilage Repair Society (ICRS) classification, grade 0 indicates normal cartilage; grade 1 indicates superficial fissuring and softening; grade 2 indicates < 50% depth to the subchondral plate; grade 3 indicates > 50% depth to the subchondral plate; and grade 4 indicates penetration into the subchondral plate.

Knees were evaluated using a GE Signa Excite 1.5-Tesla MR scanner with a superficial knee Q-coil (General Electric, Milwaukee, Wisconsin, United States). The following parameters were used in the axial fat-saturation (sat) suppressed proton sequence: fraction of repetition time (TR)/fraction of time to echo (TE): 2860/48.1 ms; fraction thickness 4 mm; field of view (FOV) 16 x 16 cm; slice thickness gap: 4/1 mm and matrix 192 x 256 pixels.

### Statistical analysis

Statistical analyses were performed using the IBM SPSS version 21.0 software (IBM Corporation, Armonk, New York, United States). Descriptive statistics are presented as means, standard deviations, numbers or proportions. Comparisons of categorical variables were made using the chi-square test, while comparisons of numerical values between groups were made using the Student t test. P values less than 0.05 were considered statistically significant. The r value was used to determine the magnitude of the relationship between pairs of variables. The inter-rater reliability between the two examiners was determined using the intraclass correlation coefficient (ICC). Values greater than 0.90 indicated excellent reliability.

## RESULTS

The intraclass correlation coefficient between the two examiners was found to be 0.91, thus indicating excellent interclass reliability of the measurements. Chondromalacia was present in 448 knees (79.5%) out of the total of 562 knees (246 males and 316 females). In the CP group, 241 were right knees and 207 were left knees; and there were 40 bilateral cases. The MRI findings of the patients were grouped according to the patella types. Out of the 562 knees, 265 (50.71%) were type I patella, 195 (36.7%) were type II, 100 (12.3%) were type III and 2 (0.3%) were type IV. Table 1 summarizes the clinical and demographic findings of the two groups.

**Table 1.** Clinical and demographical features of the groups with and without chondromalacia

Gender	Variables	CP group	nonCP group	P value
	**Age (years)**	43.16 ± 13.65	50.40 ± 16.20	**0.004**
**Male** **(n = 246)**	**Patella type**			
	1	82 (41.6)	33 (67.3)	**0.009**
	2	75 (38.1)	13 (26.5)	
	3	38 (19.3)	3 (6.2)	
	4	2 (1.0)	–	
	**Lateral trochlear inclination**	26.70 ± 2.1	27.21 ± 2.0	0.071
	**Medial trochlear inclination**	19.99 ± 2.7	20.21 ± 2.4	0.675
	**Trochlear angle**	12.73 ± 1.5	12.82 ± 1.4	0.630
	**Sulcus angle**	133.18 ± 5.1	134.88 ± 5.6	0.157
	**Patellar tilt**	11.26 ± 1.6	10.96 ± 1.3	0.318
	**Patella angle**	143.16 ± 4.2	141.85 ± 4.6	0.113
	**Insall-Salvati index**	1.09 ± 0.7	1.02 ± 0.2	0.136
**Female** **(n = 316)**	**Age (years)**	46.78 ± 13.17	52.38 ± 12.81	**0.001**
	**Patella type**			
	1	114 (45.4)	36 (55.4)	0.223
	2	86 (34.3)	21 (32.3)	
	3	51 (20.3)	8 (12.3)	
	4	–	–	
	**Lateral trochlear inclination**	26.89 ± 2.1	27.13 ± 2.2	0.274
	**Medial trochlear inclination**	19.80 ± 2.4	19.55 ± 2.4	0.355
	**Trochlear angle**	12.88 ± 1.4	12.90 ± 1.8	0.749
	**Sulcus angle**	131.83 ± 5.6	133.62 ± 5.6	0.127
	**Patellar tilt**	11.53 ± 1.4	11.10 ± 1.4	0.131
	**Patella angle**	142.85 ± 4.2	142.18 ± 4.8	0.079
	**Insall-Salvati index**	1.08 ± 0.6	1.04 ± 0.1	0.563
**Total** **(n = 562)**	**Age (years)**	51.42 ± 10.9	27.62 ± 4.8	**< 0.001**
	**Gender**			
	Male	177 (39.5)	69 (60.5)	**< 0.001**
	Female	271 (60.5)	45 (39.5)	
	**Patella type**			
	1	196 (42.8)	69 (60.5)	**0.005**
	2	161 (35.9)	34 (29.8)	
	3	89 (19.9)	11 (9.6)	
	4	2 (0.4)	0 (0)	
	**Lateral trochlear inclination**	23.49 ±2.3	27.00 ± 1.9	**0.018**
	**Medial trochlear inclination**	19.95 ± 2.5	19.66 ± 2.4	0.282
	**Trochlear angle**	12.80 ± 1.3	12.81 ± 1.4	0.743
	**Sulcus angle**	132.51 ± 5.4	132.84 ± 5.4	0.521
	**Patellar tilt**	11.28 ± 1.4	11.58 ± 5.0	0.522
	**Patella angle**	142.66 ± 4.2	143.35 ± 4.3	0.127
	**Insall-Salvati index**	1.07 ± 0.6	1.06 ± 0.1	0.791

CP = chondromalacia patella. The data are shown as n (%).

Statistically significant differences in patella type and LTI angles were found between the groups (P < 0.05). The prevalences of the different patella types in the CP group (n = 448) were type I (n = 196), type II (n = 161) and type III (n = 89) (Table 2). In the male population, the presence of CP showed a significant correlation with patella type (P < 0.05), particularly type II patella (38.1%) and type III patella (19.3%). In the female population, there was no significant correlation between patella type and presence/absence of CP. Overall, presence of CP showed a significant correlation with patella type (P < 0.05), particularly type II patella (35.9%) and type III patella (19.9%) (n = 562) (Table 2).

**Table 2.** Incidence of chondromalacia patella among the patients, according to patella type and gender

	Variables	Type 1	**Type 2** ^*^	**Type 3** ^*^	Type 4	P value
**Male** **(n = 246)**	CP nonCP	82 (71.3) 33 (28.7)	75 (85.2) 13 (14.8)	38 (92.7) 3 (7.3)	2 (100.0) -	**0.009** ^*^
**Female** **(n = 316)**	CP nonCP	114 (76.0) 36 (34.0)	86 (80.4) 21 (19.6)	51 (86.4) 8 (13.6)	- -	0.223
**Total** **(n = 562)**	CP nonCP	196 (74.0) 69 (26.0)	161 (82.6) 34 (17.4)	89 (89.0) 11 (11.0)	2 (100) 0 (0)	**0.005** ^*^

CP = chondromalacia patella. The data are shown as n (%). *Presence of CP and patella type showed a significant correlation

The LTI was significantly lower in the CP group than in the nonCP group (P < 0.05). It was 23.49° ± 2.3° in the CP group and 27.00° ± 1.9° in the nonCP group.

There was a moderate positive correlation between the severity of chondromalacia and age (r = 0.402; P < 0.001). A very weak correlation between the severity of chondromalacia and weight was also detected (r = 0.125; P = 0.03). No significant correlation was observed between the grade of chondromalacia and anatomical measurements. The correlation analyses are presented in Table 3.

**Table 3.** Correlation analyses on patients’ demographic features and morphometric measurements

Variables		Chondromalacia grade
**Age**	r	0.402^*^
	P	**0.000**
**Weight**	r	0.125^*^
	P	**0.003**
**Lateral trochlear inclination**	r	-0.026
	P	0.540
**Medial trochlear inclination**	r	-0.039
	P	0.362
**Trochlear angle**	r	-0.081
	P	0.054
**Sulcus angle**	r	0.052
	P	0.219
**Patellar tilt**	r	0.004
	P	0.925
**Patella angle**	r	0.004
	P	0.923
**Insall-Salvati index**	r	-0.077
	P	0.068

Correlation analyses denote significance.

## DISCUSSION

The main finding from this study was that in patients with anterior knee pain, the patellar morphology and lateral trochlear inclination angle may act as predictors for diagnosing CP, particularly in the early degeneration period.

CP is a condition that is characterized by softening, fraying or ulceration of the cartilage at the posterior patella, accompanied by anterior knee pain. For almost half of healthy individuals over 20 years of age and nearly every individual over 50 years of age, experience of softening of the patellar cartilage has been reported.^17,18^ The primary etiology of CP includes trauma in the knee area, repeated microtraumas, sports wounds, osteochondritis dissecans caused by vascular disorders and inflammatory diseases. CP is also frequently characterized by morphological complications of the patellofemoral joint.^17–19^

Although MRI plus clinical examination is the most appropriate approach for patients with suspected CP, studies have shown that MRI is insufficient for detecting early degenerative changes in the joint cartilage.^20,21,22^ None of the currently available imaging methods have adequate sensitivity and specificity for making an early diagnosis of CP.^20–22^ Arthroscopy is considered to be the gold standard for early detection of CP; however, it is not used in daily practice as it is an interventional procedure.^4,23^

In the current study, we observed that the incidence of CP depended on the patella type. CP was seen more commonly in patients with type II and type III patella, particularly in the male population. This finding is supported by data from previous studies.^24–26^ Arslan et al. evaluated 1,804 patients with regard to the relationship between patella type and chondromalacia. They reported that a statistically significant relationship was detected between type III patella and CP.^24^ Hayirlioglu et al. also reported that type III patella and chondromalacia were found to be statistically significantly associated.^26^

Carrillon et al. previously described a threshold LTI value for patients with patellar instability. According to their study, LTI < 11° is an excellent diagnostic test demonstrating patellar instability, with sensitivity of 0.93 and accuracy of 0.90.^10^ In our study, comparison of the relationship between the measurement methods for patellofemoral morphology showed that LTI had a significant relationship with chondromalacia (27.00° ± 1.9° in the nonCP group; 23.49° ± 2.3° in the CP group; P < 0.05). However, contrary to the findings of the current study, lack of a significant relationship between morphological measurements of the patellofemoral joint and chondromalacia has also been reported.^4,25,26^

Nonetheless, several studies support the current findings. Türkmen et al. reported that among 104 patients, the average LTI value was 21.4° ± 4.0° in chondromalacia patients and 23.2° ± 4.5° in controls.^27^ In an evaluation of trochlear morphology in a cohort of 115 patients, Duran et al. reported that the LTI value was 19.5° ± 3° in chondromalacia patients and 26.3° ± 3.1° in the controls.^4^ However, the patient group was smaller than in our study and also no significant relationship between other morphometric measurements and chondromalacia was identified in that study. Mehl et al. compared the geometry of the patellofemoral joint in 43 patients with an arthroscopically verified patellar cartilage defect and a control group of patients without this defect. Compared with the control group, the patients in the defect group had lower LTI, but without statistical significance. They reported that the LTI value was 19.1° ± 6.4° in the defect group and 20.6° ± 5.6° in the non-defect group. In that study too, the patient group was smaller than in our study.^5^

In the present study, an analysis on patients who underwent knee MRI for nontraumatic anterior knee pain showed that there was a statistically significant relationship between the patient’s age and the presence of chondromalacia (P < 0.05). In the light of the published data available, an increase in the risk of chondromalacia with increasing age is an expected finding.

The data from the current study had certain limitations. First, although the number of patients was statistically sufficient, the study group only comprised anterior knee pain patients and so the statistical values lack a control group comparison. Second, the measurements were evaluated through MRI scans alone; computed tomography scans may be more accurate for measuring the values. However, it should also be kept in mind that MRI is the gold-standard noninvasive method for evaluating chondral surfaces such as the patella. On the other hand, despite these limitations, the large sample size of our study group and the multiple comparisons among the parameters analyzed formed a strong point in our study.

## CONCLUSION

CP is highly prevalent within society and none of the imaging methods currently available can be used for making an early diagnosis of this condition. Identification of the patella type, especially in the early degenerative period, and making LTI measurements in patients with anterior knee pain and suspected CP are of great importance for correct diagnosis. The basic differential of the results from the present study was that we showed that lower LTI may be an indicator for CP in patients without complaints and may be used as an early diagnostic parameter in individuals with type II and type III patella. We recommend that detection of patella type and measurement of lateral trochlear inclination should form part of the routine work-up in assessing patients with anterior knee pain. Randomized large-scale studies including patients with no clinical complaints should be carried out to firmly establish this recommendation in the future.
